# The impact of a clinical academic nurse researcher in critical care: A 1‐year service review

**DOI:** 10.1111/jan.16367

**Published:** 2024-08-21

**Authors:** J. Ede, S. Sutherland, C. Lumley, A. Douglass, H. Walthall

**Affiliations:** ^1^ Oxford University Hospitals NHS Foundation Trust Oxford UK; ^2^ NIHR Oxford Biomedical Research Centre Oxford UK

**Keywords:** critical care, health services research, leadership, research implementation, research in practice

## Abstract

**Aims:**

To outline the activity and impact from implementing a clinical academic nurse researcher in a multi‐centre critical care unit.

**Design:**

A prospective exploratory activity audit informed by the Plan, Do, Study, Act framework.

**Methods:**

Quantitative data on clinical academic activity, from 1 April 2023 to 31 March 2024, were collected in a Microsoft Excel © [2022, version 16.66.1] spreadsheet. Activity narratives were detailed in qualitative data and the impact categorized.

**Results:**

A total of 1500 clinical academic activity hours were logged (accounting for annual leave entitlement and sickness). Of these, 973 h were directly categorized within clinical academic activity. Most frequently undertaken clinical academic activities were academic writing (17.6%), data collection/analysis (9.6%), grant and funding workup (6.8%), Intensive Care Unit service development (6.6%), clinical activity (5.2%) and local level capability and capacity (4.9%) and other supportive tasks such as administration, unscheduled meetings, critical planning time and peer support (38%). Improvements broadly mapped onto five healthcare improvement domains; organizational, process design, data optimization and utilization, evidenced‐based practice, and patients/staff impact.

**Conclusions:**

Our data indicate system mechanisms afforded by the clinical academic role that have not been explored within the contemporary literature. A nursing clinical academic demonstrates impact across the broad organization whilst increasing the visibility of nursing work and the potential for system resilience. In conclusion, our service review underscores the transformative potential of clinical academics in shaping the future of healthcare. Utilizing their expertise and contributions paves the way for innovation, excellence and sustainability in patient care.

**Impact:**

This review has provided clarity about clinical academic activity of a nurse researcher during the first year. There is international impact of this work for both clinical academics who may be evaluating such roles and healthcare management developing similar roles locally.

**Patient and Public Contribution:**

No Patient or Public Contribution.

**Why is this research needed?**

- Patients have better outcomes when cared for in research‐affiliated healthcare organizations.
- There continues to be a lack of wider understanding of how Nursing, Midwifery, and Allied Health Professionals clinical academic roles improve patient outcomes.
- Measuring the impact of these roles is challenging, and there needs to be a greater understanding of the services they provide.

**What are the key findings?**

- Most frequently undertaken clinical academic activities were academic writing (17.6%), data collection/analysis (9.6%), grant and funding workup (6.8%), ICU service development (6.6%), clinical activity (5.2%) and local level capability and capacity (4.9%) and other activities such as administration, unscheduled meetings, critical planning time and peer support (38%).
- Improvements broadly mapped onto five main improvement domains; organizational, process design, data optimization and utilization, evidenced‐based practice and patients/staff impact.
- Outputs from clinical academic work packages included research awareness, knowledge translation, direct patient impact, practice changes and project refinement.

**How should the findings be used to influence policy/practice/research/education?**

- This work provides valuable insights into the impact of a clinical academic nurse researcher in a critical care setting.
- The key findings and impact of the clinical academic nurse researcher role include quantifiable and diverse activities, impact on improvement domains, visibility of nursing work, system resilience and organizational benefits.
- This work demonstrates the need for routine investment in such roles for the organizational benefits they bring to the healthcare system.

**What does this paper contribute to the wider global clinical community?**

- This work provides useful insights for the international clinical academic community and critical care.
- It indicates ways in which other clinical academics may be able to evaluate their roles and demonstrate service, patient and staff impact in their own healthcare context.
- Resilience engineering may be a framework with which to evaluate these roles in the future.

## 1 INTRODUCTION

International healthcare organizations in Germany, United Kingdom (UK), North America and Finland have shown improved performance metrics when they are active participators in research (Clarke & Loudon, 2011; Henshall et al., 2021; Jonker et al., 2020; Jonker & Fisher, 2018; Selby & Autier, 2011). These performance measures can include higher quality of care scores (Jonker & Fisher, 2018), improved mortality in conditions such as cancer (Downing et al., 2017) or felt more widely across the organization with a happier workforce (Intensive Care Society, 2016; Selby & Autier, 2011). In UK National Health Service (NHS) settings, clinical research has measurable positive effects on staff and organizational performance, despite being a small proportion of the overall activity (Henshall et al., 2021).

In 2017, Health Education England highlighted a need to develop a workforce that embraces research and innovation to maximize patient and staff benefits (Health Education England, 2018). More recently, the Chief Nursing Officer strategic plan for research emphasized the need to utilize the research potential of the nursing workforce (National Health Service England and NHS Improvement, 2021). To enable this, the largest research funding body within the UK, the National Institute for Health and Care Research, has recognized a need to create more accessible funding for underrepresented healthcare professionals such as nurses (National Institute for Health and Care Research, 2022).

In UK healthcare settings, clinical academics combine clinical practice with research‐related activities (Vassie et al., 2020) and are well established in medicine. These roles can be seen across diverse professional groups such as physiotherapy, dietetics, pharmacy and nursing (Henshall et al., 2021; King et al., 2023; Newington et al., 2021; Trusson et al., 2019). Clinical academic nursing roles, whilst rare, are not specific only to the UK. Internationally, nursing clinical academics may be referred to as nurse scientists in the United States (Birkhoff et al., 2020) or clinical chairs in Australia (Dunn & Yates, 2000). Despite the different titles, they perform similar roles with the view to increasing research capability and capacity and growing the clinical evidence base for care within different areas of clinical nursing such as cancer, neonates (Gerrish & Chapman, 2017), palliative care and critical care (Coad et al., 2019).

## 2 BACKGROUND

Critical care is a clinical environment well suited to accommodate nursing research and academics due to the breadth of research opportunities. For instance, during the COVID‐19 pandemic, critical care research was pivotal to its effective management. Multi‐centre, international trials such as the RECOVERY study found an associated reduction in mortality with the use of steroids in ventilated patients (The RECOVERY Collaborative Group, 2021). This phenomenal piece of work demonstrated the power of research which changed the global approach to patient care in just nine months by identifying viable treatment options for critically unwell patients infected with coronavirus.

It is a UK healthcare standard that Intensive Care Unit (ICU) nurses should complete national competencies, some of which relate to research, literature exploration and evidence‐based practice as part of their ICU education (Critical Care Networks, 2015). It is also a requirement for critical care areas to engage in research and is a theme within UK‐based critical care national service guidelines (Guidelines for the Provision of Critical Care Services‐GPICS) (ICS, 2022; Intensive Care Society, 2016). Earlier document versions focus on activity that predominantly support clinical trials or aligning ICUs with a university research department (Intensive Care Society, 2016). More recent guidelines, however, suggest that engaging staff with research not only improves ICU performance but is also associated with lower burnout rates among intensive care nursing staff (ICS, 2022). Interestingly, Australia have integrated nursing research into their ICU workforce plan earlier and more comprehensively than the UK. These guidelines recommend that each critical care unit should have a lead nurse to focus on nursing research (Australian College of Critical Care Nurses, 2016).

It is crucial to evaluate the clinical academic nursing roles to ensure value for money and propagate diverse positions that provide nursing expertise. Currently, outcome measures often centre around improved patient outcomes, quality of care, safety (Carrick‐Sen & Moore, 2019), internships, publications, research funding and impact on teamwork (Henshall et al., 2021). The scope of these roles is developed according to the need of the local patient and staff population that they serve (Henshall et al., 2021), and role heterogeneity facilitates a research focus which is reflective of this. Commissioners of healthcare demand evidence of translational research demonstrated through impact measures and improvements to care, patient outcomes or service processes (Coad et al., 2019). However, measuring impact can also be challenging and complex (Newington et al., 2023), underpinning a necessity to robustly record and evaluate the services nursing clinical academics provide.

## 3 THE STUDY

### 3.1 Aims

To outline the activity and impact from implementing a clinical academic nurse researcher in a multi‐centre critical care unit.

## 4 METHODS

### 4.1 Design

A prospective exploratory audit and service review of nursing clinical academic activity, informed by the Plan, Do, Study, Act (PDSA) audit framework (HQIP, 2020) was undertaken. The Plan and Do PDSA phase within this work relates to the development, planning and implementation of the clinical academic role. The ‘Study’ phase relates to the planned clinical academic exploratory audit and service review data collection. The ‘Act’ phase within the PDSA cycle will be reflected in the recommendations which arise from this work. An inductive approach was employed to organically map the clinical activity undertaken within this new role and allocate these to broader activity themes.

### 4.2 Clinical academic nurse researcher role description (PDSA Phase: Plan and Do)

This clinical academic position was the first one advertised and employed within the local Trust. The position sits within the Trust's research infrastructure which includes the Director of Research and Innovation, Deputy Director of Research and Divisional Research Lead. The post is 1 whole time equivalent (WTE) and is allocated 300 h annual leave per year (8/52 weeks) plus 3–4 weeks sickness or carers leave (4/52). The final hours total for a 37.5‐h week means 1500 annual hours to be audited. The clinical academic role was performed by a doctorally prepared registered nurse who was also a Chartered Human Factors and Ergonomics specialist. The role provided cover across both intensive care sites, and this was not pre‐defined within the job description but was flexible depending on priorities and unit requirements for research support and education.

### 4.3 Site

The local NHS Trust constitutes three tertiary referral hospitals and one smaller hospital with 1500 beds and serves a population of over 600,000. The general, multilevel adult ICU consists of two units across two sites, has a critical care bed capacity of 48 and hosts the clinical academic role.

### 4.4 Data Collection (PDSA Phase: Study)

Over the first year of the role, clinical academic activity data were collected between 1 April 2023 and 31 March 2024 by the clinical academic. Clinical academic activities were directed based on job description, local needs analysis and Trust priorities. Categories of activities were not pre‐defined prior to the data collection, as this was an inductive exploratory approach. It was anticipated that some activities of the role would be similar to that of the research impact framework developed by Newington and colleagues (Newington et al., 2023) but that further specialized activities may also emerge as data were collected. A two‐phased approach to categorizing activities was employed. First, raw clinical activities were captured and categorized descriptively, for example, collecting nursing care metrics. These were then refined as the data collection progressed and higher‐level activity categories were identified, for example, data optimization and visualization. The data collected were presented to staff within the department for feedback and assessment of accuracy.

Tasks that constituted clinical activities have been defined in File S1. Each clinical academic activity had characteristics recorded such as date, time, duration, description, people involved, impact and any outputs detailed in a Microsoft Excel © [2022, version 16.66.1] spreadsheet. A qualitative narrative surrounding each activity was documented to add further details to the interaction or work stream. Role outputs, which refer to the tangible and measurable results, achievements or deliverables associated with a specific job or position within an organization, were also documented within the qualitative data. Several episodes of hours audited may contribute to one impact outcome. Some activities during the role were not directly categorized as clinical academic activity and were categorized as supportive tasks including administrative tasks, travel time, responding to emails, professional conversations, peer support and unscheduled meetings.

### 4.5 Ethical considerations

This paper has been reported against the SQUIRE checklist (File S2). This work was supported and authorized by the senior nursing team, no ethical approvals were required, and no staff or patient data were collected.

### 4.6 Analysis

All audit data were entered and then cleaned within the Excel spreadsheet. This included amalgamating certain activities to sit under a broader activity categorization. It was important to give higher‐level categorizations of activity for reporting and summarizing. Descriptive statistics were completed, and frequency of activity is presented as numbers and percentages. Qualitative narratives were collected and pragmatically analysed. As the aim of the analysis was not to generate theory but to describe clinical academic only, therefore, a descriptive content analysis (rather than an interpretive thematic analysis) approach was taken.

## 5 RESULTS

**FIGURE 1:**
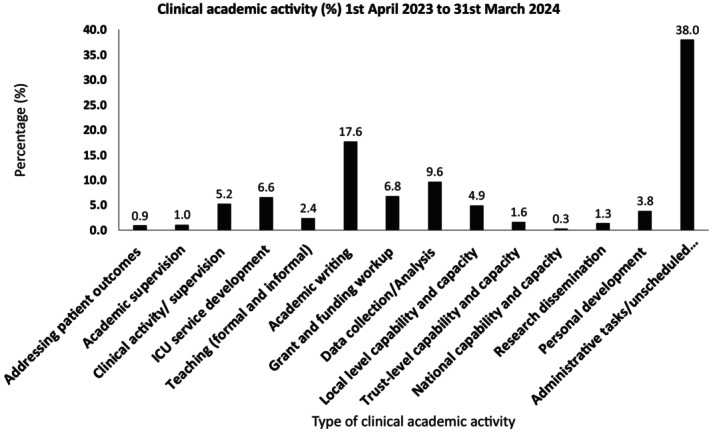
Percentage contribution of clinical academic activities over the 12‐month service period.

A total of 1500 clinical academic activity hours were logged between 1 April 2023 (accounting for annual leave entitlement and sickness) and 31 March 2024 (PDSA Phase: Study). Of these, 973 h were directly categorized within clinical academic activity. Most frequently undertaken clinical academic activities were academic writing (17.6%), data collection/analysis (9.6%), grant and funding workup (6.8%), ICU service development (6.6%), clinical activity (5.2%) and local level capability and capacity (4.9%) and supportive tasks such as administrative tasks/unscheduled meetings (38%). Full clinical academic activities are detailed in Figure 1.

### 5.1 Improvement domains

Clinical academic activities are mapped onto five main improvement domains: organizational, process design, data optimization and utilization, evidenced‐based practice, and patients/staff impact and are detailed in Table 1.

**TABLE 1:** Evidence of improvement domains and impact.

Improvement domain	Evidence of impact
Organizational	Supporting trust‐wide research capacity and capability NMAHP mentorship, teaching and coaching Facilitating internal (local Trust) and external (national) collaborations Projects delivering on trust‐wide quality priorities
Process design	Integrating human factors methodologies into clinical governance approaches such as Safety‐II Introduction of novel methods and skills such as Functional Resonance Analysis Method, Cognitive Task Analysis, Link Analysis Contributing to safety/adverse event analysis through specialist knowledge and skills Input into the design of clinical process, quality improvement projects and supporting local business cases
Data optimization and utilization	Improving the clinical utilization and access to data Developing strategies to measure nursing workload Efficient utilization of data to inform care Disseminating data from ICNARC reports (infographics)
Evidenced‐based practice	Research drop‐in sessions Education and teaching Reinforcing working relationship with library services Rapid research reviews
Patient and staff benefits	Staff development through career opportunities Research secondment and local research infrastructure Clinical supervision Implementation and sustainability of direct patient care improvements

#### 5.1.1 Organizational impact

Through the established local and a trust‐wide research network (Director of Nursing Research and Innovation and Divisional Research Leads), the clinical academic role had organizational impact by contributing to the Trust's broader vision of formalizing Nursing, Midwifery, and Allied Health Professionals (NMAHP) research opportunities. Impact was derived from practical tasks, such as participating in the research mentorship system accessible to early career researchers and delivering teaching sessions for the Trust's research courses. For NMAHPS undertaking research at a local level or developing future research fellowships/grant proposals, the position was a source of advice and expertise. Coaching was also a significant aspect of the role, where the academic provided practical support to NMAHP staff on external funding application processes (document reviews) and how best to maximize their career opportunities, making them a desirable and fundable applicant.

The academic role also created opportunities for internal and external collaborations. Local collaborations were organically formed by working across departments with teams such as resuscitation specialists. This resulted in co‐delivering projects that addressed Trust‐wide quality priorities such as recognizing and responding to clinical deterioration through employing electronically captured safety huddles. External links that benefited the Trust were created and fostered with Human Factors and Ergonomics specialists in industry, researchers, statisticians and Higher Education Institutions (HEI) nationally. Importantly, the clinical and HEI links were beneficial in terms of resources, increasing clinical access to academic expertise and facilitated an education/research/clinical communication loop. These broad collaborations increased the local research capacity and capability by bringing in project opportunities, expertise and increased funding credibility.

#### 5.1.2 Process design

Specialist research interests and knowledge of the clinical academic coupled with being a chartered Human Factors and Ergonomics specialist facilitated the introduction of novel research methods and skills into the clinical environment. These included process mapping, Functional Resonance Analysis Methods, Cognitive Task Analysis, ethnography and interviewing techniques. Organizational and staff exposure to a broad spectrum of analysis methods creates an ability to inspect and learn from a wider range of clinical issues and potentially reveals new learning. Being clinically affiliated fostered a close collaboration with local and divisional Clinical Governance teams. This allowed the clinical academic to utilize their specialist knowledge, contributing to the analysis of local and Trust safety/adverse events such as ward patient deterioration events. The impact of this was to facilitate learning and broader organizational knowledge. Similarly, affiliation with the direct clinical team meant that the academic was able to provide support and expertise on Quality Improvement (QI) projects such as addressing sedation holding compliance and contributing to business case development by sourcing data and evidence to support the bid.

#### 5.1.3 Data optimization and utilization

Enhancing the clinical utilization of and access to data was a priority workstream within the clinical academic role. Creating strategies to better utilize data (existing and newly generated) by integrating this into daily work afforded clinical staff an oversight of their care and the ability to benchmark their performance. To gain a more nuanced understanding of the ICU workload (which could indicate a strained system functioning at the limits of capacity), monthly care metrics were collected which detailed lung protective strategy compliance, sedation holds missed or delivered and the number of incident reports across the month. Specific nursing measures of workload, such as delayed patient positioning opportunities, were also collated and was data not previously utilized. This gave a more comprehensive overview of ICU performance and workload when triangulated across several system elements. Identifying and disseminating data that were already available such as Intensive Care National Audit and Research Centre (ICNARC) reports were done by creating more accessible ways to disseminate the information such as infographics, translating this knowledge to a breadth of healthcare professionals.

#### 5.1.4 Evidenced‐based practice

Impact was created by reinforcing the existing evidenced‐based care approach within the local ICU. Informal research drop‐in sessions (online, weekly for 1 h) were established, and staff were encouraged to attend these if they wanted to discuss essays, papers, clinical competencies, research careers or find out information about research being undertaken within the local unit. Sessions were chaired by a number of invited guests such as the Divisional Research Lead, Clinical Governance, Clinical Research Network Nurses, and an Outreach Librarian to create accessibility to a breadth of expertise within research and to further embed this into the workforce.

The academic supported ICU staff to complete their evidenced‐based ICU national competencies, complete literature reviews and access research by creating strong links between the library service and the critical care unit. Library teaching sessions were integrated into existing education strategies (Tea Trolley Teaching), and staff were able to register remotely for the library service to increase uptake and use of this resource. To increase the familiarity of evidence, staff were emailed a Rapid Research Review based on any current literature that was interesting or challenged local practice. These were abridged versions of original research papers that expanded on difficult statistical concepts and would usually include a visual abstract to further increase accessibility.

#### 5.1.5 Patients and staff impact

The clinical academic role demonstrated an impact on both staff and patients. A research infrastructure created education and development opportunities for critical care nursing staff, including a fully funded research secondment programme offering mentorship and flexibility to explore their own research interests. This growing infrastructure created the opportunity to develop a portfolio of in‐house ICU research that was ultimately more locally driven and able to explore the use of different methods rather than only being able to support clinical trials. Examples of such studies included examining ICU nurse workload and safety huddles. The clinical academic also gave clinical staff practical advice and support when planning and undertaking their own QI and service evaluation projects, fostering a continuous and strong improvement culture. The academic post was also pivotal in the implementation and sustainability of direct patient care improvements, including patient diaries, and patient/relative surveys.

### 5.2 Demonstrable role outputs

Clinical academic activities led to a total of *n* = 78 demonstrable outputs (measurable results, achievements or deliverables). Commonest outputs related to building research capacity, education and knowledge transfer, patient care improvements and practice change. Multiple hours of clinical academic activity within each of the improvements may contribute to a single output and are detailed in Table 2.

**TABLE 2:** Clinical academic impact and demonstrable outputs.

Impact category	Frequency (*n*)
Research awareness, capability and capacity building	26
Education and knowledge translation	22
Patient impact	13
Practice change	4
Project refinement	3
Publication	2
Project refinement	2
Grant application	1
Data visualisation	1
Service development	1
Survey	1
Education priorities	1
Internship application	1
Grand total	78

## 6 DISCUSSION

This paper has described in detail the clinical academic activity of a nurse researcher in critical care. The impact of this role is evidenced at micro‐, meso‐ and macro‐levels across the organization (Ede et al., 2022) and not restricted to the single clinical area in which it sits. Service review data indicate impact (demonstrable outputs) in five key improvement domains including organizational, process design, data optimization and utilization, evidenced‐based practice, and patient and staff benefits. Moreover, our service review is the first to propose and establish connections between mechanisms that clinical academics bring to the local system. This includes highlighting the actual workload of ICU nursing staff and reinforcing the potential for system resilience within a healthcare framework.

In this review, improvement domains and categories of impact data resonate with other work examining the role of clinical academics in healthcare. Newington et al. conducted a qualitative study (*n* = 20) and interviewed research active clinicians to identify areas of direct and indirect research impact. In their study, direct impact included fellowships, publications conference presentations and indirect impact included treatment guidelines, involvement in professional bodies and peer reviewing, improved recruitment and retention of staff were frequently monitored (Newington et al., 2021). Our data also support these as core success measures of the role and include similar outputs such as knowledge translation, capacity building and practice change.

Associations between research activity and patient outcomes might be difficult to identify (Newington et al., 2021), and it is unclear if role benefits originate from the person, strategy or through patient interventions (Coad et al., 2019). Therefore, reductionist evaluation approaches may be detrimental to fully understanding their benefit, and the literature suggests that clinical academics should select meaningful metrics on which to evaluate their role which captures relational and humanistic elements (Coad et al., 2019). As described in this service review, the clinical academic role was boundary spanning (Pattison et al., 2022) and had a reach across an organization by creating cross‐departmental collaborations, national research links with other researchers, industry relationships and bridging the clinical university gap. The role fundamentally created a *‘fusion point’* for collaboration. The role assisted in fostering strong networks and partnerships which in turn brought about opportunities to collaborate on research projects, increase potential funding credibility and were essential to local research capacity and capability building.

Our data also strongly indicates that a proportion of impact is related to using and enhancing clinical access to existing data. For example, the clinical academic was able to generate a more complete and comprehensive idea of ICU nursing workload using variables which had not been identified previously such as the number of two hourly patient position changes, number of spinal rolls and the total number of patient position changes delivered across a month. The invisibility of nursing work is a phenomenon which has been described within the literature (Smith & Aitken, 2023), and nursing workload is notoriously difficult to measure (Hoogendoorn et al., 2020). The multifaceted demands and nature of nursing work, coupled with the limited availability of professionals dedicated to illuminating these aspects, result in an insufficient understanding of the broader implications of nursing work in healthcare systems. Clinical academics are perfectly placed to shine a light on the real work‐as‐done (de Carvalho et al., 2017; Hollnagel, 2015; Sujan, 2018) within healthcare and highlight the significant level of skills and demands that nurses currently operate under in order to better inform local and national policy and resource management.

Finally, the service review data indicate system mechanisms afforded by the role which have not been explored within the contemporary literature. It could be proposed that this role contributes to the resilience potential of the organization. Resilience Engineering (RE) is a concept which underpins understanding and subsequently improvement of complex systems, enabling them to adapt and adjust to challenging conditions (Anderson et al., 2016; Hollnagel, 2015). The essential resilience potentials of a system which facilitates high‐level functioning despite variable conditions include the potential to respond, learn, monitor, anticipate and coordinate (Hollnagel, 2015). This role effectively fulfils each one of these criteria through education, data visualization, identification of priorities, internship applications, practice changes, patient impact, project change, project refinement, publications, QI development, research awareness, capability and capacity building and survey development. These outputs, which are the focus of a clinical academic role, strengthen the five resilience potentials which in turn maximize health organization's ability to respond appropriately in challenging conditions. The concept of RE may be a versatile framework with which to identify clinical academic impact in future studies examining the implementation of clinical academic roles.

## 7 STRENGTHS AND LIMITATIONS

This work is a one‐year service review of a clinical academic role in ICU, which is very specific to the local context. It has been acknowledged throughout this paper that generalizing impact across all clinical academic roles is challenging. However, this work does give other clinical academics an indication of system areas within which they may want to explore their own impact and gives evidence of role outputs. We have also proposed a possible framework (or lens) within which to do this. It should also be acknowledged that the clinical academic performing the role also collected the data as this was a review of their service. Furthermore, these data do not include any feedback from staff or patients in relation to the role as this sat outside of the initial scope of the work but is a priority for future service review and priority‐setting work.

## 8 CONCLUSION

This service review has transparently described the improvement domains in which a clinical academic role can demonstrate impact, and which benefit the wider organization. The clinical academic activity data support other literature examining the role but highlights the use and accessibility of data as a fundamental work package. A novel finding from the service review data is an indication of two key mechanisms by which a clinical academic role creates impact within an organization: addressing and reducing the invisibility of nursing work and increasing the potential for system resilience. Both of these are fundamental to patient and staff safety and efficient operational functions of healthcare. It is essential that these roles are routinely invested in given the clear organizational benefits evidenced within our data.

## AUTHOR CONTRIBUTIONS

All authors have agreed on the final version and meet at least one of the following criteria (recommended by the ICMJE (http://www. icmje.org/recommendations/)): (1) substantial contributions to conception and design, acquisition of data, or analysis and interpretation of data; (2) drafting the article or revising it critically for important intellectual content.

## FUNDING INFORMATION

No funding was associated with this work.

## CONFLICT OF INTEREST STATEMENT

No conflict of interest has been declared by the author(s) in relation to this study.

### PEER REVIEW

The peer review history for this article is available at https://www.webofscience.com/api/gateway/wos/peer‐review/10.1111/jan.16367.

## Supporting information


File S1.

File S2.

## Data Availability

Data available on request from the authors.

## References

[jan16367-bib-0001] Anderson, J. E. , Ross, A. J. , Back, J. , Duncan, M. , Snell, P. , Walsh, K. , & Jaye, P. (2016). Implementing resilience engineering for healthcare quality improvement using the CARE model: A feasibility study protocol. Pilot and Feasibility Studies, 2, 1–9. 10.1186/s40814-016-0103-x 27965876 PMC5154109

[jan16367-bib-0002] Australian College of Critical Care Nurses . (2016). Workforce Standards for Intensive Care Nursing 2016 . https://www.acccn.com.au/documents/item/542

[jan16367-bib-0003] Birkhoff, S. D. , Nair, J. M. C. , Monturo, C. , Molyneaux, D. , Rochman, M. F. , Sawyer, A. M. , & Moriarty, H. (2020). Increasing nursing research capacity: The roles and contributions of nurse scientists within healthcare systems in the greater Philadelphia region. Applied Nursing Research, 55, 151288. 10.1016/j.apnr.2020.151288 32471724

[jan16367-bib-0004] Carrick‐Sen, D. , & Moore, A. (2019). Improving care and outcome through NMAHP research‐focused clinical academic roles‐an international perspective. International Journal of Practice‐Based Learning in Health and Social Care, 7(2), II–VI. 10.18552/ijpblhsc.v7i2.648

[jan16367-bib-0005] Clarke, M. , & Loudon, K. (2011). Effects on patients of their healthcare practitioner's or institution's participation in clinical trials: A systematic review. Trials, 12, 16. 10.1016/S0140-6736(10)61045-8 21251306 PMC3036633

[jan16367-bib-0006] Coad, J. , Manning, J. , Mills, E. , Semple, C. , & McMahon, A. (2019). Capturing the real impact of clinical academics in practice. International Journal of Practice‐Based Learning in Health and Social Care, 7(2), 47–56. 10.18552/ijpblhsc.v7i2.647

[jan16367-bib-0007] Critical Care Networks . (2015). National Competency Framework for registered nurses in adult critical care step 1 competencies critical care networks‐National Nurse Leads. Critical Care Networks‐National Nurse Leads, 1–78. https://www.cc3n.org.uk/uploads/9/8/4/2/98425184/01_new_step_1_final__1_.pdf

[jan16367-bib-0008] de Carvalho, P. V. , Righi, A. W. , Huber, G. J. , Lemos, C. D. , Jatoba, A. , & Gomes, J. O. (2017). Reflections on work as done (WAD) and work as imagined (WAI) in an emergency response organization: A study on firefighters training exercises. Applied Ergonomics, 68, 28–41. 10.1016/j.apergo.2017.10.016 29409645

[jan16367-bib-0009] Downing, A. , Morris, E. J. A. , Corrigan, N. , Sebag‐Montefiore, D. , Finan, P. J. , Thomas, J. D. , Chapman, M. , Hamilton, R. , Campbell, H. , Cameron, D. , Kaplan, R. , Parmar, M. , Stephens, R. , Seymour, M. , Gregory, W. , & Selby, P. (2017). High hospital research participation and improved colorectal cancer survival outcomes: A population‐based study. Gut, 66(1), 89–96. 10.1136/gutjnl-2015-311308 27797935 PMC5256392

[jan16367-bib-0010] Dunn, S. V. , & Yates, P. (2000). The roles of Australian chairs in clinical nursing. Journal of Advanced Nursing, 31(1), 165–171. 10.1046/j.1365-2648.2000.01248.x 10632805

[jan16367-bib-0011] Ede, J. , Garry, D. , Barker, G. , Gustafson, O. , King, E. , Routley, H. , Biggs, C. , Lumley, C. , Bennett, L. , Payne, S. , Ellis, A. , Green, C. , Smith, N. , Vincent, L. , Holdaway, M. , & Watkinson, P. (2022). Building a Covid‐19 secure intensive care unit: A human‐centred design approach. Journal of the Intensive Care Society, 24, 71–77. 10.1177/17511437221092685 36860555 PMC9204129

[jan16367-bib-0012] Gerrish, K. , & Chapman, H. (2017). Implementing clinical academic careers in nursing: An exemplar of a large healthcare organisation in the United Kingdom. Journal of Research in Nursing, 22(3), 214–225. 10.1177/1744987116689133

[jan16367-bib-0013] Health Education England . (2018). Clinical Academic Careers Framework: A framework for optimising clinical academic careers across healthcare professions HEE Clinical Academic Careers Framework Background and Context . http://www.nihrtcc.nhs.uk/intetacatrain/copy_of_Medically_and_Dentallyqualified_Academic_Staff_Report.pdf

[jan16367-bib-0014] Henshall, C. , Kozlowska, O. , Walthall, H. , Heinen, A. , Smith, R. , & Carding, P. (2021). Interventions and strategies aimed at clinical academic pathway development for nurses in the United Kingdom: A systematised review of the literature. Journal of Clinical Nursing, 30, 1502–1518. 10.1111/jocn.15657 33434295

[jan16367-bib-0015] Hollnagel, E. (2015). RAG—Resilience analysis grid. Resilience Engineering in Practice: A Guidebook, 275–295.

[jan16367-bib-0016] Hoogendoorn, M. E. , Margadant, C. C. , Brinkman, S. , Haringman, J. J. , Spijkstra, J. J. , & de Keizer, N. F. (2020). Workload scoring systems in the intensive care and their ability to quantify the need for nursing time: A systematic literature review. International Journal of Nursing Studies. Elsevier Ltd, 101, 103408. 10.1016/j.ijnurstu.2019.103408 31670169

[jan16367-bib-0017] HQIP . (2020). A guide to quality improvement tools. Healthcare Quality Improvement Partnership, 1(12), 1–30.

[jan16367-bib-0018] ICS . (2022). Guidelines for the Provision of Intensive Care Services. VERSION 2.1 .

[jan16367-bib-0019] Intensive Care Society . (2016). Guidelines for the Provision of Intensive Care Services . https://www.ficm.ac.uk/sites/default/files/gpics_ed.1.1_‐_2016_‐_final_with_covers.pdf

[jan16367-bib-0020] Jonker, L. , & Fisher, S. J. (2018). The correlation between National Health Service trusts clinical trial activity and both mortality rates and care quality commission ratings: A retrospective cross‐sectional study. Public Health, 157, 1–6. 10.1016/j.puhe.2017.12.022 29438805

[jan16367-bib-0021] Jonker, L. , Fisher, S. J. , & Dagnan, D. (2020). Patients admitted to more research‐active hospitals have more confidence in staff and are better informed about their condition and medication: Results from a retrospective cross‐sectional study. Journal of Evaluation in Clinical Practice, 26(1), 203–208. 10.1111/jep.13118 30784152

[jan16367-bib-0022] King, E. , Cordrey, T. , & Gustafson, O. (2023). Exploring individual character traits and behaviours of clinical academic allied health professionals: A qualitative study. BMC Health Services Research, 23(1), 1025. 10.1186/s12913-023-10044-2 37741969 PMC10517465

[jan16367-bib-0023] National Health Service (NHS) England and NHS Improvement . (2021). Chief nursing officer for England's strategic plan for research .

[jan16367-bib-0024] National Institute for Health and Care Research . (2022). NIHR Equality, Diversity & Inclusion Strategy 2022–2027 .

[jan16367-bib-0025] Newington, L. , Alexander, C. M. , & Wells, M. (2021). Impacts of clinical academic activity: Qualitative interviews with healthcare managers and research‐active nurses, midwives, allied health professionals and pharmacists. BMJ Open, 11(10), e050679. 10.1136/bmjopen-2021-050679 PMC8499282 34620661

[jan16367-bib-0026] Newington, L. , Wells, M. , Begum, S. , Lavender, A. J. , Markham, S. , Tracy, O. , & Alexander, C. M. (2023). Development of a framework and research impact capture tool for nursing, midwifery, allied health professions, healthcare science, pharmacy and psychology (NMAHPPs). BMC Health Services Research, 23(1), 1–15. 10.1186/s12913-023-09451-2 37138350 PMC10157965

[jan16367-bib-0027] Pattison, N. , Deaton, C. , McCabe, C. , Coates, V. , Johnston, B. , Nolan, F. , Whiting, L. , & Briggs, M. (2022). Florence Nightingale's legacy for clinical academics: A framework analysis of a clinical professorial network and a model for clinical academia. Journal of Clinical Nursing, 31(3–4), 353–361. 10.1111/jocn.15756 33797144

[jan16367-bib-0028] Selby, P. , & Autier, P. (2011). The impact of the process of clinical research on health service outcomes. Annals of Oncology. Elsevier Masson SAS, 22, 5–9. 10.1093/annonc/mdr419 22039145

[jan16367-bib-0029] Smith, D. , & Aitken, L. M. (2023). Rethinking the problem of clinically deteriorating patients: Time for theory‐informed solutions. Australian Critical Care, 36, 925–927. 10.1016/j.aucc.2023.09.001 37716883

[jan16367-bib-0030] Sujan, M. (2018). A safety‐II perspective on organisational learning in healthcare organisations: Comment on “false dawns and new horizons in patient safety research and practice”. International Journal of Health Policy and Management. Kerman University of Medical Sciences, 7, 662–666. 10.15171/ijhpm.2018.16 PMC6037496 29996587

[jan16367-bib-0031] The RECOVERY Collaborative Group . (2021). Dexamethasone in hospitalized patients with Covid‐19. New England Journal of Medicine, 384(8), 693–704. 10.1056/nejmoa2021436 32678530 PMC7383595

[jan16367-bib-0032] Trusson, D. , Rowley, E. , & Bramley, L. (2019). A mixed‐methods study of challenges and benefits of clinical academic careers for nurses, midwives and allied health professionals. BMJ Open, 9(10), 1–9. 10.1136/bmjopen-2019-030595 PMC6797317 31594886

[jan16367-bib-0033] Vassie, C. , Smith, S. , & Leedham‐Green, K. (2020). Factors impacting on retention, success and equitable participation in clinical academic careers: A scoping review and meta‐thematic synthesis. BMJ Open, 10(3), e033480. 10.1136/bmjopen-2019-033480 PMC7170560 32213518

