# Supplementary material for: The impact of a clinical academic nurse researcher in critical care: A 1‐year service review
Source: J Adv Nurs. 2024 Aug 21;81(4):1806–14. doi: 10.1111/jan.16367 (PMC11896936; doi:10.1111/jan.16367)
Supplement: Supplementary file 1 — File S1. [file JAN-81-1806-s002.docx]

## Supplementary File 1. Clinical Academic Activity Definitions

| Activity | Definitions |
| --- | --- |
| **Clinical activity/ supervision** | - Co-ordinating/bedside shifts - Intra-hospital transfers - Bedside teaching - Education shifts - Evidenced Based Rounds - Tea Trolley Teaching |
| **Grant and funding workup** | - Drafting and writing grants - Collaborations and knowledge exchange - Signposting to grant/funding opportunities - Identifying grant/funding opportunities suitable for individual needs |
| **Research dissemination** | - Conferences - Teaching - Generating social media content - Webinars - Social media promotion for research |
| **Trust-level capability and capacity** | - Local conferences - Trust research mentorship - Supervising - Social media promotion for research training opportunities |
| **Addressing clinical processes** | - Improvement and clinical governance - Safety and adverse events learning - Guideline reviews - Process designs - Application of Human Factors methods - Integration of research with safety trends |
| **Addressing patient outcomes** | - Practice changes - Supporting the use of evidenced base care bundles - Implementation of patient care strategies (sedation holding/patient diaries) |
| **Data collection and analysis** | - Designing data collection tools - Piloting data collection strategies - Collecting data - Data curation and governance - Care record reviews/interviews/observations - Analysis (Quant/Qual/HF) - Data visualisation |
| **National capability and capacity** | - Developing or informing national research opportunities |
| **Teaching (formal and informal)** | - Presentations - Conferences - Classroom teaching - Research drop-in clinic teaching - Direct care skills - Use of excel/Nvivo/SPSS training - Critical appraisal skills |
| **ICU service development** | - Clinical governance processes - Protocol/guideline development - Being on an interview panel - Delivering on recruitment initiatives |
| **Local level capability and capacity** | - Developing local research opportunities - Creating and facilitating networking opportunities - Signposting to expertise (local/national/HEI) |
| **Personal development** | - Courses - Training - Personal mentorship and coaching |
| **Academic writing** | - Academic writing planning - Academic drafting - Submissions of manuscripts to peer reviewed journals |
| **Academic Supervision** | - Essay/dissertation reviews - HEI ethics applications - Research skills - Self-promotion opportunities |
| **Supportive Tasks** | - Travel time - Responding to emails - Professional conversations - Peer support - Administrative tasks - Supportive tasks/unscheduled meetings - Reviewing and locating relevant literature - Critical ‘thinking time’ - Identifying and screening literature - Ad Hoc coaching |
